# Review: Balancing Selection for Deleterious Alleles in Livestock

**DOI:** 10.3389/fgene.2021.761728

**Published:** 2021-12-03

**Authors:** Martijn F. L. Derks, Marije Steensma

**Affiliations:** ^1^ Animal Breeding and Genomics, Wageningen University and Research, Wageningen, Netherlands; ^2^ Topigs Norsvin Research Center, Beuningen, Netherlands

**Keywords:** balancing selection, overdominance, loss-of-function, artificial selection, animal breeding

## Abstract

Harmful alleles can be under balancing selection due to an interplay of artificial selection for the variant in heterozygotes and purifying selection against the variant in homozygotes. These pleiotropic variants can remain at moderate to high frequency expressing an advantage for favorable traits in heterozygotes, while harmful in homozygotes. The impact on the population and selection strength depends on the consequence of the variant both in heterozygotes and homozygotes. The deleterious phenotype expressed in homozygotes can range from early lethality to a slightly lower fitness in the population. In this review, we explore a range of causative variants under balancing selection including loss-of-function variation (i.e., frameshift, stop-gained variants) and regulatory variation (affecting gene expression). We report that harmful alleles often affect orthologous genes in different species, often influencing analogous traits. The recent discoveries are mainly driven by the increasing genomic and phenotypic resources in livestock populations. However, the low frequency and sometimes subtle effects in homozygotes prevent accurate mapping of such pleiotropic variants, which requires novel strategies to discover. After discovery, the selection strategy for deleterious variants under balancing selection is under debate, as variants can contribute to the heterosis effect in crossbred animals in various livestock species, compensating for the loss in purebred animals. Nevertheless, gene-assisted selection is a useful tool to decrease the frequency of the harmful allele in the population, if desired. Together, this review marks various deleterious variants under balancing selection and describing the functional consequences at the molecular, phenotypic, and population level, providing a resource for further study.

## Introduction

Since the widespread use of artificial insemination in livestock, a small number of popular sires can ‘dominate’ the breeding population. Consequently, the effective populations size (Ne) decreases, causing deleterious alleles to rise in frequency. Hence, the inherited defects generally involve unique ‘founder’ variants (Fasquelle et al., 2009). Founder variants are variants observed at high frequency in a specific population that derive from a single influential ancestor. Generally, these type of deleterious alleles are purged from a population by (natural) selection. This purging is efficient for dominant deleterious alleles that lower the fitness of heterozygous animals. However, as a function of the low frequency, recessive deleterious alleles are generally masked from natural selection by a dominant non-deleterious allele, and will therefore be easily passed on into the next generation.

In population genetic theory, the amount of deleterious alleles arising from mutation is expected to be equal to the amount of deleterious alleles purged by (natural) selection, resulting in a mutation - selection balance (Charlesworth and Willis 2009). Hence, harmful alleles are kept in balance at a low frequency in the population, due to the elimination through purifying selection and the occurrence of new variants (Old, 1993). However, recent studies have revealed various harmful alleles present at a moderate to high frequency in the population (Georges et al., 2019). This high frequency of recessive harmful alleles can be a result of genetic drift, i.e. the random fluctuations in the numbers of gene variants in a population, especially in populations with low effective population size (Bosse et al., 2019). However, genetic drift alone cannot explain the high frequency of some relatively high frequency variants identified. Hence, a second explanation stems from variants exhibiting antagonistic pleiotropic effects, driving the frequency of such variants (Hedrick, 2015). The concept of antagonistic pleiotropy involves a trade-off between a beneficial effect on one trait and a detrimental effect on another trait, caused by a single variant (Hedrick, 1999). One common mechanism of antagonistic pleiotropy is heterozygous advantage, also called overdominance. Heterozygous advantage depends on an interplay between the strength of the advantage in heterozygotes and the negative consequences in homozygotes, leading to a balance between purifying selection against mutant homozygotes, and positive selection on heterozygotes (Hedrick, 2015). In addition, the advantage might be affected by numerous population genetic factors including the allele frequency, the sex, and the selection goal. Together, balancing selection refers to selective processes by which alleles are maintained in a population at frequencies larger than expected from genetic drift alone (Siewert and Voight, 2017).

Due to the wide increase of genomic and phenotypic data in livestock breeding, numerous examples of deleterious variants under balancing selection have been published. However, this mechanism is likely far more common than expected, as many deleterious variants remain “hidden” in the population (Charlesworth, 2006). The artificial selection for the variants is performed based on the advantage in heterozygotes, while the negative consequence in homozygotes is only expressed at higher frequencies. In addition, the molecular mechanisms underlying balancing selection are often poorly understood (Fijarczyk and Babik, 2015). Most examples refer to a single allele that exhibits antagonistic effects on distinct traits, usually resulting from a dominant effect in heterozygotes, while a complete loss of function of a gene leads to the deleterious phenotype in homozygous mutants. However, also multiple variants in close linkage disequilibrium (LD) have been described having separate effects on different traits (Charlesworth, 2006). Therefore, a similar effect can be caused by two variants that are closely linked on the same haplotype, each individually affecting a different trait. However, it remains a large challenge to disentangle the individual variant effects given that the variants are strongly linked (Li et al., 2015). Finally, providing an insight in the management of these variants in populations and selection schemes would be useful for a healthy population in the long term (Georges et al., 2019).

The aim of this literature review is to get an insight in known variants under balancing selection in livestock and possibly their underlying molecular mechanisms. In addition, this review aims at discussing several aspects including the management of these variants in populations and selection schemes.

## Harmful Alleles Under Balancing Selection in Livestock

This review focusses on harmful alleles under balancing selection that have been discovered in various livestock species including pig (*Sus scrofa*), cow (*Bos taurus*), sheep (*Ovis aries*), chicken (*Gallus gallus*) and the horse (*Equus caballus*). Deleterious alleles under balancing selection can have different modes of inheritance: dominant or recessive modes are most common. Additional types of inheritance include sex-linked (affecting one of the sex chromosomes), codominant (wherein the alleles of a gene pair in a heterozygote are fully expressed), and polar overdominance (where the phenotype is dependent on the parent of origin). In this section, we describe in detail harmful alleles under balancing selection, the affected traits, and the molecular background. The molecular background of the alleles under balancing selection includes a wide variety of types from single nucleotide polymorphism (mostly affecting a single amino acid) to complex large structural changes. Overall, most variants described lead to a loss of function of the affected gene, resulting in a functional change or the absence of the protein product. In this section we review the various types of molecular mechanism and the functional consequence of the alleles under balancing selection per species. An overview of the examples described in this review are presented in Table 1.

**TABLE 1:** An overview of the 18 examples of alleles with a heterozygote advantage and a homozygote disadvantage in livestock species.

Species (breed)	Trait (References)	Gene(s) involved	Type of variant	Inheritance	Heterozygote advantage	Homozygote disadvantage
Pig (Large White)	Fetal lethality Derks et al. (2018)	*BBS9, BMPER*	Frameshift (deletion)	Autosomal recessive	Increased feed intake and growth	Fetal death
Pig (Large White)	Leg weakness Matika et al. (2019)	*MSTN*	Stop-gained	Autosomal recessive	Increase in muscle depth and decrease in fat depth	Leg weakness syndrome
Pig (Finnish Yorkshire)	Immotile short-tail sperm Sironen et al. (2012)	*SPEF2*	Frameshift (insertion)	Autosomal recessive	High female litter size	Male infertility
Pig (Pietrain, Landrace)	Malignant hyperthermia Vogeli et al. (1994)	*RYR1*	Missense	Autosomal recessive	High lean meat content	Pale soft exudative meat
Cattle (Belgian Blue)	Crooked tail Fasquelle et al. (2009)	*MRC2*	Frameshift and missense	Autosomal recessive	Enhanced muscular development	Crooked tail syndrome
Cattle (Nordic Red)	Embryonic lethality Kadri et al. (2014)	*RNASEH2B*	Frameshift (deletion)	Autosomal recessive	High milk yield	Embryonic lethal
Cattle (Belgian Blue and Shorthorn)	Roan coat Seitz et al. (1999)	*KITLG*	Missense	Autosomal Co-dominant	Roan phenotype	White heifer disease
Sheep	Mastitis Rupp et al. (2015)	*SOCS2*	Missense	Autosomal recessive	Higher body weight and milk production (also in homozygotes)	Mastitis
Sheep	Fecundity Hanrahan et al. (2004)	*BMP15, GDF9*	Stop-gained and missense	BMP15: X-linked GDF9: Autosomal recessive	Increase ovulation rate and litter size	Female infertility
Sheep (Soay)	Polledness Wiedemar and Drögemüller (2015)	*RXFP2*	Frameshift (insertion)	Autosomal recessive	Higher overall fitness	Ho^P^/Ho^P^ males: lower reproductive success
	Ho^+^/Ho^+^ males: lower survival				
Sheep	Chondrodysplasia Beever et al. (2006)	*FGFR3*	Missense	Autosomal recessive	Larger animals	Spider lamb syndrome
Sheep	Callipygous phenotype Kim et al. (2004)	*DLK1 - PEG11*	Intergenic	Polar overdominance	Muscular hypertrophy	-
Chicken (Wyandotte)	Rose comb Imsland et al. (2012)	*MNR2, CCDC108*	Inversion	Autosomal dominant	Rose comb	Male infertility
Chicken	Creeper Jin et al. (2016)	*IHH*	Deletion	autosomal dominant semi-lethal	Short legs	Lethal before hatch
Horse (Appaloosa, Knabstrupper)	Leopard complex spotting Bellone et al. (2010)	*TRPM1*	Frameshift (insertion)	Autosomal incompletely dominant	Leopard complex spotting	Congenital night blindness
Horse (American Paint)	Frame pattern Ayala-Valdovinos et al (2016)	*EDNRB*	Missense	Autosomal dominant	Frame overo	Lethal white foal syndrome
Rabbit	Dwarfism Carneiro et al. (2017)	*HMGA2*	Deletion	Autosomal recessive	Dwarfism	Lethal
Rabbit	English Spotting Fontanesi et al. (2014)	*KIT*	Regulatory	Autosomal recessive	English spotting	Dilated (“mega”) cecum and ascending colon

The table also includes one example of polar-overdominance, of which the phenotype depends on the parental origin of the variant.

### Pig (*Sus scrofa*)

In a Large White breed, a recessive lethal deletion within the *BBS9* gene is assumed to cause fetal lethality in mutant homozygotes (Figure 3A). The deletion suggests antagonistic pleiotropic effects on fertility and growth, as it indicates an increase infeed intake and growth in heterozygotes. It is likely that the *BBS9* gene is under balancing selection, due to the moderate carrier frequency of 10.8% and the appearance of positive phenotypic effects in carriers (Derks et al., 2018). The fetal lethality in pigs is caused by a 212-kb deletion in the Bardet-Biedl Syndrome 9 (*BBS9*) gene. The deletion causes skipping of 4 coding and 4 3′UTR exons, resulting in direct splicing from exon 19 to exon 28. The deletion leads to a frameshift that results in a truncated protein due to a premature stop codon. This truncated BBS9 protein has lost its function (causing increased growth rate in heterozygous pigs). The 212-kb deletion affects a region which contains *BMPER cis*-regulatory elements and therefore reduces the expression of the *BMPER* gene which seems to cause fetal mortality in mutant homozygous pigs (Derks et al., 2018).

**FIGURE 1:**
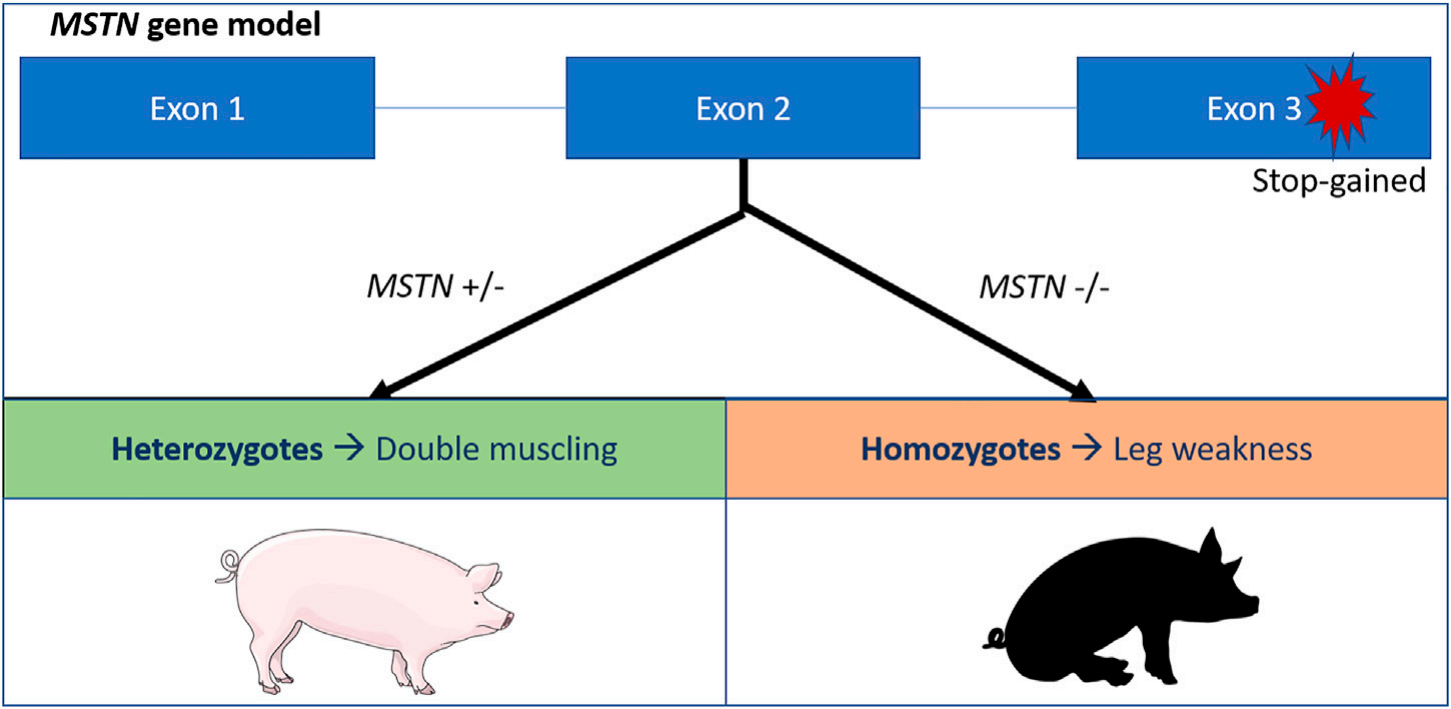
Schematic overview of the *MSTN* stop gained variant in pigs (Matika et al., 2019). The stop codon affects the third exon of the *MSTN* gene, leading to a premature stop codon and a loss of function of the myostatin protein. Heterozygotes exhibit the double muscling phenotypes, while homozygotes suffer from leg weakness.

A second deleterious allele in pigs has been described causing leg weakness syndrome (lameness) in mutant homozygotes. The allele increases muscle depth in heterozygotes explaining the high frequency (22%) in the Large White breed (Matika et al., 2019). Leg weakness in pigs is caused by a stop-gained variant replacing the glutamate with a stop codon (p.Glu274*). This results in a loss of function of the *MSTN* gene, that causes hypertrophy and leading to the so called ‘double muscling’ phenotype (Matika et al., 2019) (Figure 1).

Older examples in pig include a disruptive intronic 9-kb insertion variant in the *SPEF2* gene causing immotile short-tail sperm (ISTS) which leads to male infertility, but increased litter size in females (Sironen et al., 2012). A second example is a missense variant in the RYR1 gene (p.Arg615Cys) inducing malignant hyperthermia (MH) in homozygotes, but higher lean meat content in heterozygotes (Salmi et al., 2010).

### Cow (*Bos taurus*)

Belgian Blue cattle are popular for their high muscularity. A variant in the *MRC2* gene causes enhanced muscular development in heterozygotes, but negative effects on fitness in homozygotes (Fasquelle et al., 2009). Affected individuals have the so called ‘crooked tail syndrome’. All cases show, next to a deviation of the tail, growth retardation, abnormal shape of the skull and extreme muscular hypertrophy. Due to the heterozygote advantage, the variant was positively selected resulting in a carrier frequency of 25% in Belgian Blue cattle (Fasquelle et al., 2009). The crooked tail syndrome in cattle is caused by a c.1906T>C transition within exon 3 of the *MRC2* gene. This variant causes an amino acid substitution (p.Cys636Arg) within the third C type lectin-like domain (CTLD3). This leads to oligomerization of the Endo180 protein, subsequently leading to a loss of function. (Sartelet et al., 2012). A second variant caused by a 2-bp deletion (c.2904-2905delAG) results in a frameshift that causes a premature stop codon in the *MRC2* gene (p.Gly934*). The deletion in the *MRC2* gene causes a truncated Endo180 protein that results in the loss of function of *MRC2* (Fasquelle et al., 2009).

Next to that, Kadri et al. (2014) found a deletion comprising the *RNASEH2B* gene that results in embryonic lethality in mutant homozygous Nordic Red cattle. In carriers, positive effects on milk yield and composition were discovered. This resulted in the high carrier frequency of 13, 23 and 32% in Danish, Swedish and Finnish Red cattle, respectively. This high carrier frequency contributed to the decreased fertility in these breeds over the last years (Kadri et al., 2014). Embryonic lethality in cattle is caused by a 660-kb deletion encompassing 4 genes, including the ribonuclease H2 subunit B (*RNASEH2B*) gene. This deletion results in loss of the whole *RNASEH2B* gene, causing embryonic lethality. The deletion shows positive effects on milk yield in heterozygous cattle, but the molecular mechanism behind this advantage remains unknown (Kadri et al., 2014).

In addition, in Belgian Blue and Shorthorn cattle, a missense variant in the *KITLG* gene, resulted in females lacking the Müllerian ducts, causing sterility. The disease is also known as the White Heifer disease, as 90% of the cases show a completely white phenotype (Figure 3D) (Reissmann and Ludwig, 2013). Heterozygotes have the so called ‘roan phenotype’, an intermingled color with some white spotting, resulting in the blue and red phenotype in Belgian Blue and Shorthorn cattle, respectively. After elimination of ‘White Heifer animals’ and progeny testing of sires, the harmful allele frequency was reduced (Charlier et al., 1996). Roan coat in cattle is caused by an amino acid substitution of alanine to asparagine at amino acid 193 (p.Ala193Asp) (Seitz et al., 1999).

### Sheep (*Ovis aries*)

In different sheep breeds, variants are found in the *BMP15* and *GDF9* genes underlying the “Fecundity” phenotype that cause increased ovulation rate and litter size in female heterozygotes but impaired oocyte development and maturation in homozygous females, resulting in female infertility (Javanmard et al., 2011). Due to the heterozygote advantage, the carrier frequency for variants in the *GDF9* and *BPM15* gene reached high frequencies in many sheep breeds (Demars et al., 2013). Fecundity in sheep is caused by different types of variants (mostly missense) in the growth differentiation factor 9 (*GDF9*) gene and/or *BMP15* gene. Both genes belong to the transforming growth factor beta (TGFβ) superfamily and the *GDF9* is an autosomal gene, while *BMP15* is X-linked. Five missense variants have been described in *GDF9*. In *BMP15*, however, six missense, two stop-gained, a small deletion variant, and a complex rearrangement have all been described (Nicholas, 2021).

**FIGURE 2:**
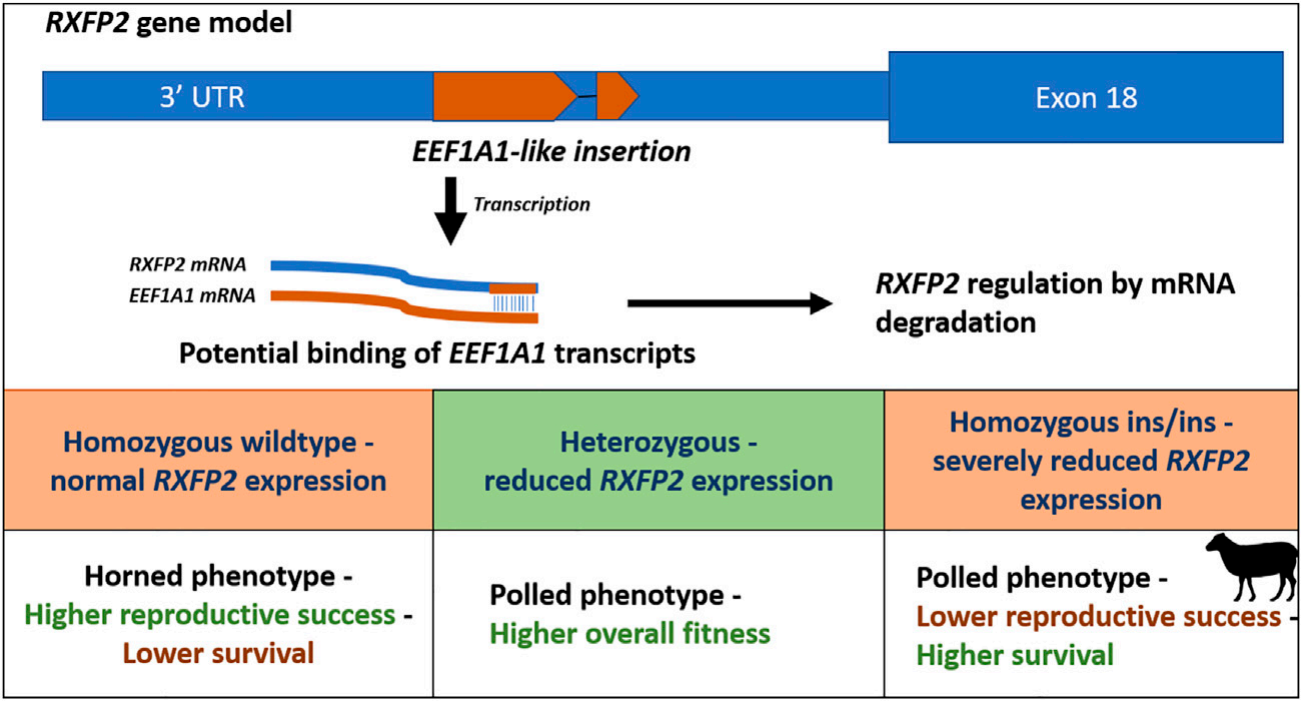
Schematic overview of the polledness trait in Soay sheep (Johnston et al., 2013; Wiedemar and Drögemüller, 2015). The polledness phenotype is associated with a *EEF1A1*-like insertion in the 3′UTR of the *RXFP2* gene. The insertion potentially leads to *RXFP2* post transcriptional regulation by binding *EEF1A1* transcripts (caused by double stranded RNA degradation). Homozygous wildtype sheep have horns and higher reproductive success but lower survival, whereas homozygous ins/ins sheep exhibit lower reproductive success but higher survival. Heterozygotes exhibit the highest overall fitness underlying balancing selection.

A second unique form of pleiotropy is the ‘polledness’ trait found in Soay sheep, as both the recessive and dominant allele show negative effects on fitness in homozygous males. Homozygous males for the Ho+ allele, which confers larger horns, have lower survival while homozygous males for HoP allele, which confers smaller horns, have lower reproductive success (Johnston et al., 2013). In contrast, heterozygous males show a higher overall fitness due to increased survival and reproductive success. However, in females, both alleles do not have any effect on fitness. The allele frequency of the mutant HoP allele stabilized close to the equilibrium frequency, because of opposite selection on reproduction and survival (Johnston et al., 2013). The ‘polledness’ trait in sheep is caused by an 1833-bp insertion in the 3′UTR region of the relaxin/insulin-like family peptide receptor 2 (*RXFP2*) gene. This insertion adds an RNA antisense sequence of *EEF1A1* to the 3′end of *RFXP2* transcripts. Likely, *EEF1A1* transcripts bind to the 3′ end of the *RXFP2* mRNA, resulting in double stranded mRNA that will be degraded, reducing levels of the *RXFP2* protein product (Wiedemar and Drögemüller, 2015) (Figure 2).

**FIGURE 3:**
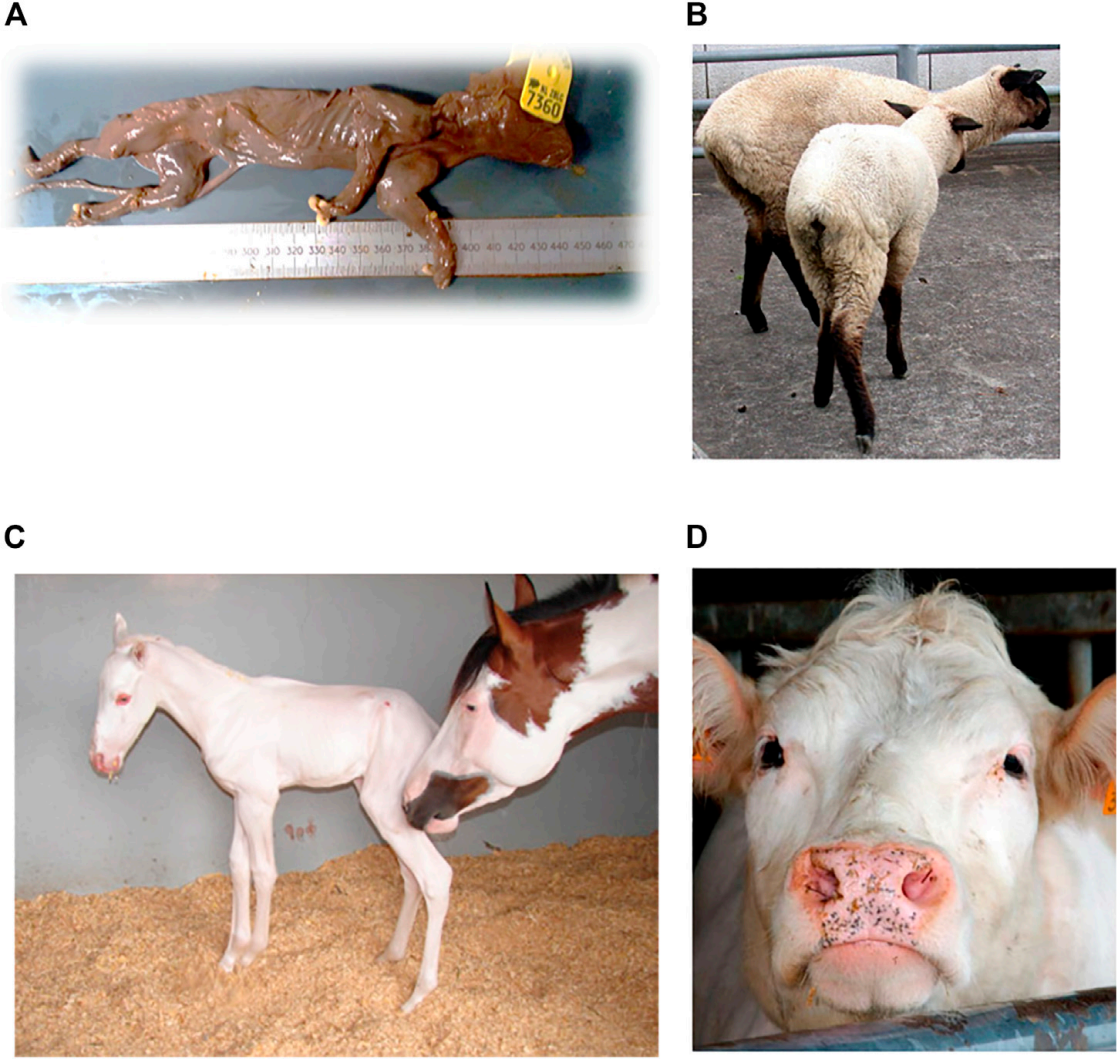
Examples of **(A)** Mummified piglet resulting from a large deletion in pigs, figure from (Derks et al., 2018). **(B)** Ovine hereditary chondrodysplasia, also known as the Spider Lamb Syndrome, figure from (Thompson et al., 2008) **(C)** Lethal white foal syndrome, figure from (Ayala-Valdovinos et al., 2016) **(D)** White heifer disease, figure from (de Meuter, 2010).

Furthermore, a relatively old autosomal recessive disease, described by Cockett et al. (1999) causes hereditary chondrodysplasia in the Suffolk breed. Affected lambs exhibit the Spider Lamb Syndrome (SLS) (Cockett et al., 1999), a severe syndrome affecting growth of cartilage and bone (Figure 3B). In contrast, heterozygotes for the mutant allele are larger-framed sheep, with increased bone length and meat yield, possibly explaining the high frequency in this breed (Smith et al., 2006). Chondrodysplasia in sheep is caused by a p.Val700Glu substitution affecting the highly conserved tyrosine kinase domain ll, resulting in the loss of function of the *FGFR3* gene and thereby producing skeletal overgrowth (Beever et al., 2006).

Lastly, a single missense variant in the *SOCS2* gene (p.R96C) was found with positive effects on growth and milk production in sheep (Rupp et al., 2015). However, homozygous animals are also more susceptible to develop mastitis compared to wildtype and heterozygous animals. More specifically, heterozygous animals exhibit similar milk yield and fat content compared homozygotes, while growth is significantly higher in homozygotes compared to heterozygotes.

### Chicken (*Gallus gallus*)

Strong artificial selection for the rose comb phenotype, affecting the *MNR2* gene, severely affects fertility in homozygous males (poor sperm motility). This results in male infertility when the sperm competes with that from heterozygous males (which is problematic if pools of semen are used) (Imsland et al., 2012). Rose comb in chicken is caused by a 7.38-Mb inversion with breakpoints located approximately at 16.50 Mb and 23.88 Mb within the homeodomain protein (*MNR2*) gene on chicken chromosome 7. This inversion causes a delocalization of the *MNR2* gene, leading to a misexpression of the gene during comb development. The rearrangement disrupts the gene *CDCC108* (located at breakpoint 23.88), responsible for male infertility in homozygous animals (Imsland et al., 2012).

Secondly, the creeper phenotype, caused by a deletion of the Indian hedgehog (*IHH*) gene, resulting in pronounced shortness of the extremities in heterozygotes was observed in chicken. Homozygous mutants generally die on the fourth day of embryonic development (Jin et al., 2016). The short legged phenotype was a popular characteristic in various breeds, explaining the high frequency of this allele in a wide range of breeds (Jin et al., 2016).

### Horse (*Equus caballus*)

In the horse, various coat color phenotypes are associated with negative effects on fitness. In the Appaloosa and Knabstrupper, heterozygous animals for a variant in the *TRPM1* gene show a leopard complex spotting phenotype, a desired phenotype for breeders. However, the mutant homozygotes exhibit congenital stationary night blindness (Bellone et al., 2013). Leopard complex spotting in horses is caused by a 1378-bp insertion in intron 1 of the *TRPM1* gene. The insertion is a long terminal repeat (LTR) of an endogenous retrovirus (ERV) disrupting the expression of *TRMP1* by premature poly-adenylation preventing translation of the last amino acids (Bellone et al., 2013).

The “frame overo pattern” is another type of coat color in which horses exhibit white spots over their body. The frame overo pattern allele has a dominant mode of inheritance, but leads to the lethal white foal syndrome in homozygotes (Figure 3C). Affected foals have a near or completely white coat and lack enteric ganglion cells; intestinal agangliosis (Bellone, 2010), usually resulting in death within the first 24 h of life. The harmful allele is under balancing selection, as carriers have a phenotypic advantage, leading to a frequency up to 21.6% in the American Paint breed (Badial et al., 2018). The frame pattern in horses is caused by a di-nucleotide substitution c.353TC>AG within the first exon in the endothelin receptor type B (*EDNRB*) gene. This dinucleotide change causes an amino-acid substitution of isoleucine to lysine at amino acid 118 (p.Ile118Lys). This amino acid substitution to a charged amino acid is located within the first transmembrane domain of the 7-transmembrane domain G protein coupled receptor but how it affects function remains to be determined (Bellone, 2010).

### Rabbit (*Oryctolagus cuniculus*)

In rabbits the *KIT* gene was found to be responsible for the English spotting coat colour in heterozygous *En/en* individuals. However, homozygous *En/En* animals exhibit a dilated (“mega”) cecum and ascending colon leading to problems with the digestive system especially during flare-ups of the disease (Fontanesi et al., 2014). Another example in rabbits includes a 12.1 kb deletion affecting the *HMGA2* gene leading to dwarfism in heterozygotes, but leading to a lethal phenotype in homozygotes (Carneiro et al., 2017).

### Major Histocompatibility Complex

A gene family that is under balancing selection in livestock is the major histocompatibility complex (MHC) class genes. Heterozygotes usually exhibit increased disease resistance, while homozygosity in the MHC region will likely cause increased susceptibility to disease due to a lower diversity at the peptide-binding region, resulting in lower effectiveness in recognizing pathogens (Hedrick, 1998). Hence, genetic diversity in the MHC class gene family increases fitness, but scientists are only beginning to understand the molecular mechanisms driving this fitness advantage (Hammer et al., 2020).

## Common Trait Selection Affects Similar Genes Across Livestock Species

Because of similar selection goals in livestock, genes can influence the same traits in different livestock species, leading to comparable phenotypes. However, variants affecting the same gene in different livestock species do not necessarily show antagonistic pleiotropic effects in either species.

### Double Muscling

One key example is the ‘double muscling’ phenotype caused by variants in the *MSTN* gene described in pig, cattle, poultry, and sheep (Nicholas, 2021). The double-muscle trait is characterized by an increase in muscle mass, resulting in significantly higher meat yield (Aiello et al., 2018). The double muscling phenotype provides obvious advantages for animal breeders, but generally coincides with major drawbacks like the larger incidence of calving difficulties in cattle, and leg-weakness in pigs.

### Coat Color

Genes affecting coat color give rise to comparable phenotypes in livestock. For example, mutations in the *EDNRB* gene, causing lethal white foals in horses (Bellone, 2010), have also been reported in sheep and chicken (Nicholas, 2021). More specifically, a 110-kb deletion in the *EDNRB* gene causes hypopigmentation and a megacolon, known as Waardenburg syndrome type 4A in sheep. Sheep homozygous for this deletion have a white coat and blue eyes and die shortly after birth, because of intestinal obstruction similar to the aganglionosis present in lethal white foals (Lühken et al., 2012). The Waardenburg syndrome is very similar to the lethal white foals in horses, as both homozygous animals express a white coat, intestinal obstruction or aganglionosis, and die shortly after birth. The *KIT* ligand is a second gene affecting coat color in a wide range of breeds and species (Reissmann and Ludwig, 2013). In addition to the roan coat color in cattle, also in horses an analogous phenotype associated with the *KIT* gene has been reported (Marklund et al., 1999).

### Polledness

In sheep, homozygote males for both HoP and Ho+ have lower fitness and heterozygote males have an overall higher fitness (Wiedemar and Drögemüller, 2015). In goats, interestingly, the polled intersex syndrome (PIS) is described. This syndrome is a disorder of sexual development showing an association with the polled phenotype and intersexuality. PIS is caused by a 10-kb deletion and a 480-kb insertion containing the genes *ERG* and *KCNJ15* leading to a complex rearrangement (Simon et al., 2020). In cattle, polledness is caused by complex duplication variants on BTA1 that leads to differences in gene expression (Wiedemar and Drögemüller, 2015).

### Other Examples

In sheep, variants in the *FGFR3* gene lead to the spider lamb syndrome, and larger animals in heterozygotes (Beever et al., 2006). In cattle, a stop-lost variant that affects the cattle *FGFR3* gene has been reported causing a dominant form of chondrodysplasia. The variant is a *de novo* mosaic variant as the parents of the calves with chondrodysplasia were not affected (Häfliger et al., 2020).

The gene responsible for malignant hyperthermia in pigs has also been reported in both cattle (Hill et al., 2000) and horse breeds (Aleman et al., 2004). In horses, more specifically, a harmful missense variant in the American Quarter horse leads to a phenotype analogous to the hyperthermia in pigs.

## Polar Overdominance

Polar overdominance is a unique and rare type of overdominance. This phenomenon refers to heterozygous animals only expressing the phenotype depending on the parental origin of the variant.

### Callipygous Phenotype

The first discovered and most well-known type of polar overdominance is the callipyge variant in sheep. Heterozygous sheep which inherited the variant from their sire express the phenotype. The callipygous (CLPG) phenotype is characterized by muscular hypertrophy (‘double muscling’) of the hind legs. Lambs which inherited the callipygous variant from their sire show a normal phenotype at birth, but develop the callipygous phenotype from 30 till 80 days after birth (Cockett et al., 1996). The callipygous phenotype is caused by an A to G transition in the intergenic region between the *DLK1* and *GTL2* genes. This results in an increased expression of the *DLK1* and *PEG11* genes on the paternal haplotype. Hence, the variant affects *cis*-acting regulatory sequences resulting in a gain of function of *DLK1* (Freking et al., 2002).

Furthermore, the callipygous phenotype also occurs in goats, but in this breed only individuals which receive the mutated allele from their dam express the phenotype. The callipygous phenotype in goats is caused by a C to T transition (Li et al., 2009). Next to that, Kim et al. (2004) found that the polar overdominance is present in the porcine *DLK1-GTL2* region that is homologous to that of the sheep CLPG locus. Pigs who inherited the paternal *DLK1* allele 2 and the maternal *DLK1* allele 1 exhibit higher lean muscle mass and decreased fat deposition. In contrast, pigs who inherited the maternal *DLK1* allele 2 and the paternal *DLK1* allele 1 exhibit lower pre- and postnatal growth (Kim et al., 2004).

## Population Genetics and Management of Alleles Under Balancing Selection

### The Balance Between Drift and Selection

Due to the overdominance effect, the frequency of the discussed alleles can become unexpectedly high in the population. Examples have been published describing lethal alleles at a carrier frequency >10% just by drift in various livestock species (Charlier et al., 2016; Efendić et al., 2018; Derks et al., 2019a). Hence, an unusual high frequency of deleterious variants does not constitute an advantage in heterozygotes per se. Therefore, not only pleiotropic effects can lead to a moderate frequency of deleterious alleles. The frequency can also be influenced by genetic drift and the power of genetic drift heavily depends on the effective population size (Derks et al., 2019a). Leroy et al. (2013) studied the effective population size in several dog, sheep, horse and cattle breeds and found that the effective population size is small (Ne < 100) in most commercial livestock breeds. As a consequence (mildly) deleterious alleles can reach a relatively high frequency, or even become fixed in a population due to genetic drift (Leroy et al., 2013). Charlier et al., 2016. specifically studied the number and frequency of lethal alleles (the most severe type of deleterious allele) (Charlier et al., 2016). They showed that the number and frequency of these type of variants depends on several population genetic parameters including the genomic target size for lethal mutations, the rate of recessive lethal mutations in this target space, and especially the present and past effective population size. They concluded that the number of recessive lethals carried on average per individual increases with effective population size from about one for an Ne of 100 to ∼7.7 for Ne of 10.000. However, the frequency of deaths as a result of these lethal variants is nearly similar (between 1.54 and 1.73%). Together the results show that a small effective population size leads to high extinction rates of harmful alleles, but the few alleles that did not went extinct tend to rapidly spread in the population. Hence, the number of deleterious alleles that segregate in a population with low Ne is much smaller compared to high Ne populations. However, the deleterious variants that do segregate tend to segregate at higher frequency. Nevertheless, at a certain frequency, there will be a trade-off (the maximum allele frequency that can be reached by drift alone) between purifying selection and genetic drift. At this frequency, the homozygote loss will be larger than the heterozygote advantage.

**FIGURE 4:**
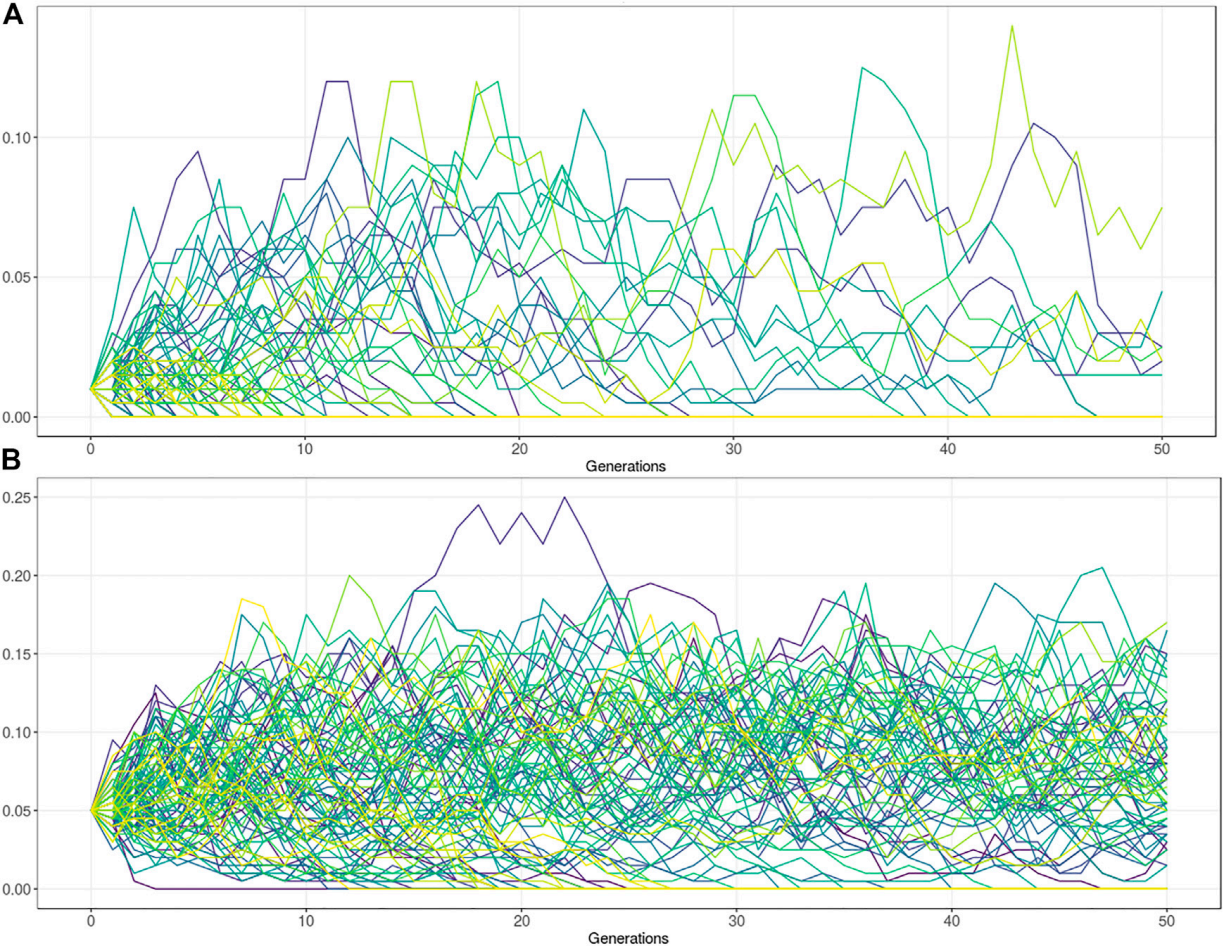
Population genetic simulations for deleterious alleles with Ne = 100. **(A)** Frequency simulation of a deleterious allele at starting frequency 1%. The fitness of the homozygous BB genotype = 0, with equal fitness for the AB and AA genotype (fitness = 1). Figure shows that the allele is lost in the vast majority of the populations after 50 generations. The maximum frequency reached by drift is approximately 10%. **(B)** Frequency simulation of a deleterious allele with the fitness of the homozygous BB genotype = 0, and the AB genotype has a 10% fitness advantage over the homozygous AA genotype (AB = 1, AA = 0.9). Figure shows that the allele is lost in about one third of the populations after 50 generations. In two third of the populations the allele remains at a relatively stable equilibrium between 5–15% allele frequency.

The variants under balancing selection are subject to a complex interplay between the advantage in heterozygotes and the disadvantage in homozygotes. Artificial selection has had major consequences for these types of variants. By using influential sires in the population, novel variation can rise in frequency relatively rapidly. Strong selection and the use of influential sires (the influential sire effect) can cause undetected, undesirable deleterious alleles in the population to spread rapidly (Leroy and Baumung, 2011). It generally also reduces genetic diversity by the exclusion of other males. The influential sire effect reduces the effective population size which subsequently affects the power of drift effects in the population. In small Ne populations most deleterious variants that arise tend to disappear relatively quickly. However, a small subset of variant can spread rapidly throughout the population. To perform such population simulation studies several tools have been published (Peng et al., 2015). The tools are useful to simulate the expected frequency of deleterious alleles given several population genetic parameters. Users can assess the impact of heterozygous advantage on the allele frequency, and the possible trait-off value at which the deleterious phenotype in homozygotes prevents further frequency increase. The tool takes a starting frequency and relative fitness of three genotype classes (AA, AB, BB). Further population parameters include the mutation rate, migration rate, effective population size, and the number of generations. Given an Ne of 100, with no advantage in heterozygotes, the maximum frequency of a lethal allele (Fitness BB = 0) is about 10%, but in the vast majority of the simulations the allele is lost after 50 generations (Figure 4A). Note, that a fitness of zero means that the animals cannot reproduce but could still result in a viable phenotype. However, if the heterozygotes (AB) have a 10% fitness advantage over homozygous wildtype (AA), the frequency stays at a relatively stable (10–15% AF) equilibrium (Figure 4B). Together this equilibrium frequency is affected by an interplay of the fitness advantage in heterozygotes, disadvantage in homozygous mutants, and the selection goal.

### Purging Strategies

After discovery, the harmful allele can be eliminated from the population, if desired. However, the rate at which a deleterious allele can be removed from a population, partly depends on the stage at which the negative consequence is expressed. For example, defects that results in distinct birth defects will be noticed sooner compared to defects that lead to early lethality. The strategy of elimination depends on the heterozygote advantage, the homozygote disadvantage and the allele frequency in the population. One efficient way to purge the variant is by eliminating all carriers and affected individuals from the population. However, depending on the allele frequency, a large part of the population will be eliminated, increasing genetic drift, and lowering the genetic diversity. Consequently, the risk of inbreeding will increase and thereby the risk of developing new deleterious variants. Alternatively, gene-assisted selection can be used to prevent matings between parents carrying the same deleterious variant (Georges et al., 2019). This method reduced the carrier frequency of ISTS in Finnish Yorkshire pigs by 30%. Next to that, gene-assisted selection has been applied to Pietrain and Landrace breeds, resulting in elimination of the variant causing PSS in these breeds (Hedrick, 2015). Sometimes, the frequency of the harmful allele will be kept at a relatively stable level in the population (as for the rose comb trait in chickens). Additionally, the harmful allele causing polledness in sheep stabilized close to the equilibrium frequency, as both the wild-type and mutant homozygote state show negative fitness effects (Johnston et al., 2013). Nevertheless, we need to keep in mind that sometimes the carrier frequency is already greatly reduced before the causative variant was discovered. For example, PSS pigs were halothane sensitive and through halothane tests these pigs were excluded from the population before DNA tests were available (Vogeli et al., 1994). Moreover, breeding objectives can differ between countries and change over time, thereby influencing the allele frequency. For example, the mutant *PLAG1* gene increases stature but has a negative effect on the fertility in cattle (Utsunomiya et al., 2017). This allele increased in frequency after a period of selection on smaller cattle, while larger cows are now preferred in some breeds and countries. In addition, the frequency of the *DGAT1* variant, which decreases protein yield and increases fat yield, shifted due to selection goal differences over time (Georges et al., 2019). For the mutant alleles that only have a sex-limited negative effect, a mating strategy can be developed to increase the number of heterozygotes with positive phenotypic effects and decrease the number of lethal homozygotes. For example, the fecundity trait in sheep is lethal in homozygous females, and therefore mutant homozygous males (aa) could be crossed with homozygous females (AA).

## Discussion

In this review, we describe various harmful alleles under balanced selection exhibiting positive selection on heterozygotes and purifying selection on homozygotes. Despite the vast increase of genomic data, the exact molecular mechanisms underlying the alleles under balancing selection remain largely unknown.

The described examples likely only present the tip of the iceberg and many alleles under balanced selection remain hidden in the population due to the low frequency or by the lack of genomic resources. Next to that, rare birth defects in livestock species are often noted as ‘weak animal’ and no further investigation to possible underlying genetic syndromes is done (Derks et al., 2019b). However, only a small subset of birth defects will be caused by recessive deleterious variants, of which an even smaller subset could constitute an advantage in heterozygotes. In addition, variants with only limited advantage in heterozygotes, but severe disadvantages in homozygotes will likely not reach moderate to high frequency in the population. Additionally, the lack of genomic tools especially for natural and small-sizes domestic populations hamper the detection of alleles under balancing selection. Nevertheless, some examples of harmful alleles under balancing selection have been described in wild populations. For example, the black coat phenotype in the Yellowstone wolf population (affecting the *CBD103* gene) leads to a higher fitness compared to homozygotes that exhibit lower recruitment and survival (Coulson et al., 2011; Hedrick, 2012). Secondly, the leopard complex spotting in horses, described in this review, was also reported in ancient wild horse populations potentially providing camouflage for predators in the snow (Pruvost et al., 2011). Alternatively, an example of a balanced lethal system is found in crested newts, where heterozygotes are viable and both homozygotes (wild-type and mutant) are lethal (Grossen et al., 2012). Though, in most cases, high frequencies of harmful alleles in small threatened wild populations are likely driven by genetic drift (Trask et al., 2016).

### Heterozygous Disadvantage

Interestingly, also variants that cause a heterozygote disadvantage have been described, like bovine tuberculosis. Cattle heterozygous for the locus on *BTA6* show increased susceptibility for bovine tuberculosis in comparison with homozygotes (Tsairidou et al., 2018). However, especially for most genes related to the immune system (e.g. the major histocompatibility complex), heterozygosity and high genetic variability is often beneficial for proper defense against various pathogens (Huchard et al., 2010). For example, heterozygous leopard frogs have a higher chance of survival when infected with a fungal pathogen compared to homozygotes (Savage and Zamudio, 2011).

### Allelic Pleiotropy

**FIGURE 5:**
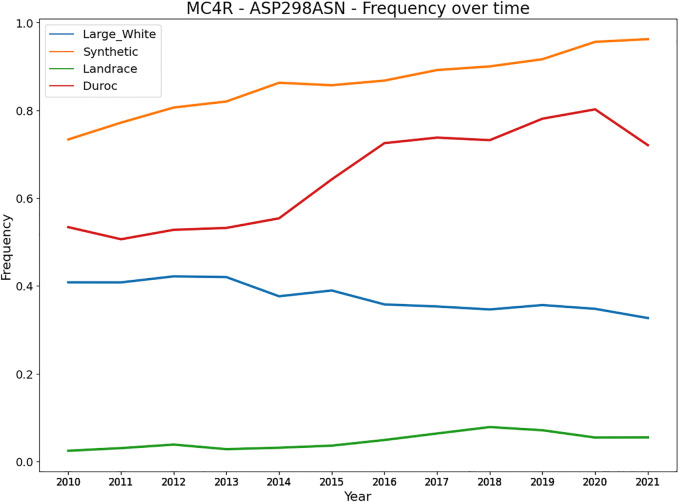
Frequency of the MC4R - Asn298 allele in four commercial pig breeds from Topigs Norsvin. The Asn298 allele is associated with more fat, higher feed consumption and faster growth, while the Asp298 allele is associated with less backfat, slower growth, and lower feed intake (Kim et al., 2000).

Besides heterozygote advantage, variants can be under balancing selection because they affect multiple traits (allelic pleiotropy) that are (negatively) correlated. Variants with a large effect on a trait likely also affect other traits, caused by (negative) trait correlations affecting similar pathways and genes that regulate multiple traits. A key example is a variant in the *MC4R* in pigs affecting growth rate, fat composition, and feed intake in different pig breeds (Kim et al., 2000). The Asp298 allele is associated with less backfat, slower growth, and lower feed intake, while the Asn298 allele is associated with more fat, higher-feed consumption, and faster growth. This variant can remain at a rather stable equilibrium in the population depending on the selection intensity of the associated traits. More specifically, we evaluated the frequency of the *MC4R* missense variant over the last decade in four commercial pig populations (Figure 5). The Large White and Landrace breed are sow breeds, with more emphasis on reproduction traits and mothering abilities, whereas the Duroc and Synthetic are boar breeds with more emphasis on growth. Figure 4 shows the frequency of the Asn298 allele (associated with more fat, higher feed consumption and faster growth). Notably, the frequency of this allele is higher (and increasing) in both boar lines (with stronger selection on growth related traits). However, for the sow lines the frequency is much lower (especially in Landrace) and remains at a more stable equilibrium likely due to lower selection intensities on growth and other production traits. An example in cattle involves four clusters of SNPs close to the genes *GPAT4, MGST1, DGAT1 and PAEP*, of which each has an allele that lowers milk and protein yield and increases fat yield. (Xiang et al., 2017).

Most variants are first identified because they exhibit a deleterious effect on a specific trait. Once identified, its consequences on another traits can be tested and quantified. However, this antagonistic effect does not immediately proof the pleiotropic effect, despite the possibility that one gene affects several traits by affecting a single or multiple pathways. Testing the effect of the variant in an different genetic context would be required to further support the pleiotropic effect.

In this review, the described variants are pleiotropic and the positive selection in heterozygotes is caused by the direct effect of the variant itself. However, a similar effect can be caused by two variants that are closely linked on the same haplotype. For example, two different variants could reside on the same haplotype, one with beneficial effects and the other one with a deleterious effect. Examples of such variants are very rare and most described variants under balancing selection exhibit antagonistic pleotropic effects. The efficiency to separate the two variants by recombination depends on the local LD and haplotype structure in the population. In livestock, LD patterns can differ greatly between different loci in the genome which partly depends on the recombination frequency (Qanbari, 2019).

### Different Types of Causative Variants and the Coding Sequence Bias

We review a wide variety of causative variants underlying heterozygote advantage phenotypes. Some variants are due to small changes (i.e., single nucleotide substitutions or small indels), while other variants include changes of structural origin. The majority of the described variants lead to a loss of function of a particular gene either by directly affecting the coding sequence of the gene, or due to changes in expression. Even if a single gene is affecting the same trait in multiple species (i.e., *MSTN* gene resulting in the double muscling phenotype) with different types of underlying variants, they overall lead to the same consequence; a loss of function of the myostatin protein.

Key examples of regulatory variants include the deletion in the *BBS9* gene in pigs and the *RXFP2* gene in sheep that reduce the expression of the downstream gene, while the inversion affecting *MNR2* in chickens leads to a misexpression of the causative gene (Hedrick, 2015; Wiedemar and Drögemüller, 2015; Derks et al., 2018). In contrast, the regulatory variant that causes the callipygous phenotype in sheep, results in increased expression of the *DLK1* gene leading to a gain of function (Freking et al., 2002). Most variants that are identified affect the coding sequence of the genes, this is partly because loss of function variants often affect the coding sequence of a gene. Nevertheless, we expect that regulatory variants are potentially equally common, but remain more challenging to identify.

### Effects in Crossbreeding

The deleterious alleles described are mostly found in purebred breeds. However, the final production animals in pigs and chicken are crossbreds between purebred breeds. These crossbreds perform better on fertility and growth traits as a result of the heterosis effect. Therefore, deleterious alleles uniquely segregating in a purebred breed will never be homozygous in crossbreds. So, the final production animals only take advantage of the pleiotropic effect of the deleterious allele (Derks et al., 2019a). Nonetheless, crossbreds can become homozygous for the mutant allele when the variant is present in both the maternal and paternal purebred breeds. Despite the limited impact on crossbreds, given that most harmful alleles are population specific, purging of deleterious variants is desired within purebreds, because of the potential economic losses, and the effect on animal welfare.

In this review, we focused on variants that exhibit heterozygous advantage but have a deleterious effect in homozygotes. We believe that with current availability of genomic and phenotypic datasets a multitude of variants will likely be discovered in the near future, giving better insights into how common these types of variants are. We believe that livestock breeds provide a key framework to study these types of variants due to the high level of genomic resources, deviating selection intensities, and increase of molecular data.
